# Advances in catalytic hydrogen storage materials: progress, breakthroughs, and challenges toward scalable applications

**DOI:** 10.3389/fchem.2026.1928154

**Published:** 2026-10-14

**Authors:** Pan Shulin, Walid Tahri, Rashid Khan, Mazen Alshaaer

**Affiliations:** 1 Key Laboratory of Yangtze Aquatic Environment, Research Base, Yibin University, Yibin, China; 2 Zhejiang Provincial Key Laboratory of Advanced Chemical Engineering Manufacture Technology, College of Chemical and Biological Engineering, Zhejiang University, Hangzhou, China; 3 Physics Department, College of Sciences and Humanities, Prince Sattam Bin Abdulaziz University, Al-Kharj, Saudi Arabia

**Keywords:** catalytic materials, hydrogen storage, metal hydrides, metal-organic frameworks, sustainable hydrogen energy

## Abstract

Hydrogen storage and release catalysts that are large-scale, efficient, and reversible provide a significant challenge for the development of long-term hydrogen energy systems (HES). This review critically examines catalytic approaches to hydrogen storage systems (HSS), including, metal hydrides, metal-organic frameworks (MOFs), covalent organic frameworks (COFs), porous materials, nanostructured catalysts, bimetallic alloys, hybrid composites, and single-atom catalysts (SACs). It evaluates how catalyst composition, structure, and active-site properties influence hydrogen activation, transport, sorption kinetics, cycling stability, and reversibility. Drawing on experimental findings and theoretical approaches, the review compares these relationships across different material classes. This comparative focus extends assessments based on individual materials or catalyst classes by identifying shared design principles and materials specific limitations. Finally, it outlines the main barriers and proposes research directions for developing sustainable, efficient, and reversible hydrogen storage.

## 1 Introduction

Due to its carbon-free use, high gravimetric energy density, and potential to enable large-scale integration of renewable energy, hydrogen is quickly becoming a crucial component of the global transition towards low-carbon energy systems. Transport, industry, and long-term energy storage are among the hard-to-electrify sectors that could benefit greatly from hydrogen as an energy carrier. However, the absence of efficient, cost-effective, affordable, and safe HS technologies is currently limiting its widespread adoption. Traditional storage methods, including high-pressure compression and cryogenic liquefaction, require substantial energy input. In addition, gaseous hydrogen has a low volumetric density and significant diffusivity, making its safe and compact storage a major challenge (Dawood et al., 2020; Nachtane and Faik, 2025; Nwokolo et al., 2026; Villanueva Martinez et al., 2025). The current techniques for storing hydrogen have their limitations; other alternatives have been proposed, with the catalytic HS approach showing great promise. To improve the efficiency of catalytic HS, designer catalysts can reduce activation energy barriers and reaction rates under mild operating conditions, enabling more efficient hydrogen absorption and desorption. Catalysts are crucial components for improving hydrogen storage capacity (HSC), enhancing reversibility, increasing energy efficiency, and ensuring long-term cycling stability. HS processes exhibit a wide range of mechanisms and properties, and materials differ significantly in their ability to achieve high HSC, rapid hydrogen sorption rates, and desirable thermodynamic behavior. As technologies for catalysts and materials engineering advances, HS could become even more useful for large-scale energy storage (Qiao et al., 2024; Scarpati et al., 2024; Yang et al., 2023).

Classical metal hydrides with relatively high HSCs, such as MgH_2_ and NaAlH_4_, have been the subject of substantial research (Ali and Ismail, 2021; Nemukula et al., 2025; Schimmel et al., 2005). However, their poor hydrogen absorption/desorption kinetics and high thermodynamic stability require high operating temperatures for hydrogen release and make them impractical for use. On the other hand, porous carbon materials and MOFs possess highly desirable properties such as very high specific surface area and highly tunable pore architectures. Their capacity to use hydrogen under ambient conditions, however, is greatly limited by weak physisorptive interactions and pore blockage by atmospheric moisture and other contaminants (Qiao et al., 2024; Sutton et al., 2024). Researchers have sought out more effective catalytic systems that can overcome thermodynamic and kinetic obstacles in order to tackle these issues.

Many new catalysts, including hybrid-composite systems, bimetallic alloys, COFs, and single-atom catalysts (SACs), have been developed (Liang et al., 2025). Their enhanced catalytic performance is attributed to synergistic effects among components, a high density of active sites, and tailored and electronic architectures (Tao et al., 2025). The development of hybrid materials with multifunctional capabilities is a recent trend (Liang et al., 2025; Wu Y. et al., 2024). SACs, with their unique catalytic functional properties and high atom-use efficiency, are particularly intriguing. Along with material development, catalytic techniques are becoming a focus of research for HS and recovery from subsurface formations as a potential alternative to material innovation for geological HS (Ershadnia et al., 2023; Cheng et al., 2025). This new approach provides potential path towards large-scale and long-term HS, complementing traditional material-based approaches.

The relationship between catalyst structure and composition, fundamental reaction pathways, and experimentally quantifiable storage performance is still poorly understood, despite significant progress in this area (Krebsz et al., 2025). This gap limits the transfer of promising laboratory-scale results to scalable storage technologies. This brief overview critically evaluates classical, advanced, and emerging catalytic materials for hydrogen storage. It then examines how theoretical and computational methods support the interpretation of experimental observations and the identification of structure-property-performance relationships. Lastly, it discusses the principal barriers to practical implementation and proposes priorities for future catalyst development.

## 2 Fundamentals of catalytic hydrogen storage (HS)

Physisorption and chemisorption are essential mechanisms governing the HS process and play a key role in the interaction of the hydrogen molecules with the storage materials (Mahmoud et al., 2025; Wen et al., 2025; Qureshi et al., 2024). The physisorption process relies on the comparatively rapid adsorption/desorption rates of a material to weakly but reversibly adsorb hydrogen onto its surface (Le et al., 2024). In most cases, this process takes place with porous materials like activated carbons, MOFs, and COFs (Qureshi et al., 2024; Osman et al., 2024a). Due to hydrogen’s weak binding energy on the adsorbent surface, its effectiveness is generally limited to low temperatures and/or high pressures (Qureshi et al., 2024; Le et al., 2024).

In complex hydride systems and metal hydrides, chemisorption occurs when hydrogen atoms form stronger chemical interactions with the host material (Ocampo et al., 2025; Zhang X. et al., 2024). While physisorption often results in faster hydrogen release rates and lower energy barriers, chemisorption offers the advantage of higher HS density (Zhang X. et al., 2024). However, this strong bonding leads to high desorption temperatures, which limits practical applications unless appropriate catalysts are introduced.

Due to the difficulties in achieving thermodynamic stability and kinetic accessibility, HS systems have garnered considerable attention (He et al., 2026; Jiang et al., 2024). By decreasing the activation energies needed for hydrogen dissociation, diffusion, and recombination, catalysts can assist in overcoming these obstacles. Transition metals such as Ni, Fe, Ti and Pd are frequently used because of their ability to dissociate hydrogen molecules and enhance hydrogen mobility within solid matrices (Ocampo et al., 2025; He et al., 2026). Additionally, advancements in nanoscale engineering have allowed for substantial improvements of catalytic characteristics through the manipulation of electronic structure, the exposure of active sites, and the expansion of surface area (Zhang X. et al., 2024; Jiang et al., 2024). The three primary stages of the catalytic HS process are as follows: adsorption/dissociation of molecular hydrogen on this catalyst surface; diffusion of atomic hydrogen into the storage medium; and reversible desorption when hydrogen is released (Jiang et al., 2024). At each stage, the catalyst’s electronic characteristics, its nature, and its interaction with the host material are crucial factors. For example, strong metal-hydrogen interactions may hinder hydrogen release, whereas overly weak interactions may enhance reversibility but reduce storage capacity.

The “spillover” effect, in which hydrogen atoms move from the metal catalyst to the support material, is another concept related to catalytic HS (Zhang W. et al., 2024). The combination of catalytic sites and the large surface area of the porous support material can be extremely beneficial in composite materials including MOFs and metal-loaded porous carbons. The impact of hydrogen spillover on the enhancement of HS at room temperature has been the subject of extensive research, despite the fact that its quantitative contribution to HS performance the function is still debatable. In general, surface chemistry, material structure, and reaction kinetics are the three fundamental pillars upon which catalytic HS rests. For next-generation catalytic systems to fulfill the needs of sustainable hydrogen energy technologies, it is crucial to understand these concepts.

## 3 Key catalytic materials for HS

The characteristics and catalytic activity of the HS material being stored have a significant impact on HSS performance (Yang et al., 2025; Liu et al., 2021; Oh et al., 2024). Reversible materials with high storage capacity, rapid hydrogen absorption and desorption rates, and reliable reversibility under operational conditions have been a focus of material development over recent decades (Peng et al., 2024). The practical value of a catalytic HS material depends on how much hydrogen it can deliver and how reliably it performs under operating conditions. To asses these properties, performance claims require quantitative measures under specified conditions. For onboard light duty vehicles, the U.S department of Energy’s 2025 (DOE) targets for a complete storage system include 5.5 wt% usable H_2_, 40 kg H_2_ m^‒3^, a minimum full flow rate of 0.02 g s^‒1^ kW^‒1^, 1,500 operational cycles, and 90% onboard delivery efficiency (U.S. Department of Energy, 2019). For comparison, a 2025 DOE assessment projected approximately 5.1 wt% and 26 kg H_2_ m^-3^ for 700 bar Type IV compressed HS, illustrating the challenge of meeting both capacity Targets at the system level (Simmons et al., 2025). When comparing catalytic materials, studies should therefore report hydrogen absorption and release temperatures and pressures, measured release rates, capacity retention during cycling, and the energy required for heating or compression. Because the system includes components the storage medium, material level capacities should not be compared directly with system-level targets. Consequently, Table 1 compares the advantages and disadvantages of the three main classes of systems, including traditional hydrides, porous adsorbents, and advanced engineered designed systems (Liu et al., 2021; Peng et al., 2024). Generally, the development of catalytic HS materials has moved toward more complex-multicomponent, and rationally architectures (Yang et al., 2025; Zhang J. et al., 2024). Despite major progress made, the simultaneous optimization of storage capacity, reaction kinetics, and reversibility under ambient conditions remains challenging. Addressing these trade-offs requires structurally tunable catalytic architectures that can promote hydrogen activation and transport without compromising stability. Figure 1 shows a classification of the main materials used for hydrogen storage and differentiates between the roles of catalysts and storage media. By facilitating hydrogen activation, transport, absorption, and release, catalysts can alleviate significant constraints, as illustrated in Figure 1.

**TABLE 1:** Benchmarking of catalytic materials for hydrogen storage: performance, mechanisms, and limitations.

Catalyst/Material type	Representative system	Hydrogen storage performance	Operation condition (PT)	Storage/Catalytic mechanism	Key advances	Limitations	Ref.
Classic metal hybrides	MgH_2_, NaAlH_4_ TiFeH_2_, NaAlH_4_	MgH_2_: ∼1.5–7.6 wt% H_2_	200 °C–400 °C, low to moderate pressure	Reversible chemisorption and hydride formation; catalytic dissociation and diffusion of hydrogen atoms	High storage density, good reversibility, operational safety	Slow kinetics, high desorption temperature, oxidation sensitivity	Scarpati et al. (2024), Nemukula et al. (2025), Schimmel et al. (2005)
Complex hydrides	LiBH_4_, NaBH_4_ Mg (BH_4_) _2_, LiAlH_4_	∼8 to 18 wt% H_2_ (theoretical)	250 °C–600 °C	Multistep decomposition of ionic/covalent hydrides; catalyst assisted destabilization	Extremely high hydrogen capacity	Poor reversibility, thermal instability, difficult regeneration	Boretti (2025), Geb et al. (2024), Kalibek et al. (2024)
Porous carbons materials	Activated carbon, CNTs, graphene, carbon aerogels	∼1 to 6 wt% H_2_	Cryogenic temperature and/or high pressure	Surface physisorption through weak van der Waals interactions	Lightweight, low cost, rapid adsorption kinetics	Low room temperature storage capacity	Rampai et al. (2024), Thomas et al. (2024)
metal‐organic frameworks (MOFs)	MOF 5, HKUST 1, UiO 66, ZIF 8	∼3 to 8 wt% H_2_	77 K; 1–100 bars	Reversible adsorption within ultrahigh-surface-area porous frameworks	Exceptional porosity, tunable pore architecture	Moisture instability, expensive synthesis, weak ambient storage	Qiao et al. (2024), Peng et al. (2024), Letwaba et al. (2024)
Covalent organic frameworks (COFs)	COF 1, COF 102, TpPa based COFs	∼2 to 5 wt% H_2_	Mostly cryogenic conditions	Hydrogen physisorption in lightweight crystalline organic networks	High tunability, low density, chemical versatility	Limited volumetric capacity and structural stability	Osman et al. (2024b), Chen et al. (2025)
Nanostructured catalysts	MgH_2_@carbon, LiBH_4_@MOFs, MgH_2_ confined in nanoporous scaffolds	∼5 to 12 wt% H_2_	150 °C–350 °C depending on hybrid and scaffold	Nanoconfinement shortens diffusion pathways and alters hydride thermodynamics hydrogen dissociation	Enhanced kinetics and reduced desorption temperature	Complex fabrication and cycling degradation (noble metals)	Geb et al. (2024), Bagyalakshmi et al. (2026)
Catalytically doped hydrides	Ti doped NaAlH_4_, Nb_2_O_5_/MgH_2_, Ni/MgH_2_ composites	∼4 to 7 wt% H_2_	200 °C–350 °C	Transition metal catalysts accelerate H_2_ dissociation and recombination reactions	Reduced activation energy and faster sorption kinetics	Catalyst agglomeration and long-term instability	Scarpati et al. (2024), Boretti (2025), Chen et al. (2025)
Single-atom catalysts (SACs)	Pt/FeOx, Ni/C, Pd based SACs	Improved hydrogen dissociation and catalytic efficiency reported	Moderate temperature operation commonly reported	Maximized atomic utilization and enhanced active sites	High catalytic efficiency and atom utilization	Stability and aggregation challenges during cycling	Liang et al. (2025), Zhang et al. (2024b)
Bimetallic/alloy catalysts	Ni Fe, Mg Ni, Pd Ag systems	Enhanced hydrogen sorption kinetics and reversibility reported	200 °C–350 °C	Synergistic electronic and catalytic interactions	Improved hydrogen uptake and release kinetics	Alloy phase segregation and degradation	Yadav et al. (2025), Wu et al. (2024b), Verma et al. (2023)
Hybrid composites	MOF carbon composites, hydride carbon hybrids, graphene composites	Enhanced kinetics and cycling stability widely reported	Conditions vary depending on composite design	Combined catalytic and porous adsorption mechanisms	Capacitance: Combined advantages of multiple materials	Synthesis complexity, scalability	Osman et al. (2024b), Bagyalakshmi et al. (2026)

**FIGURE 1:**
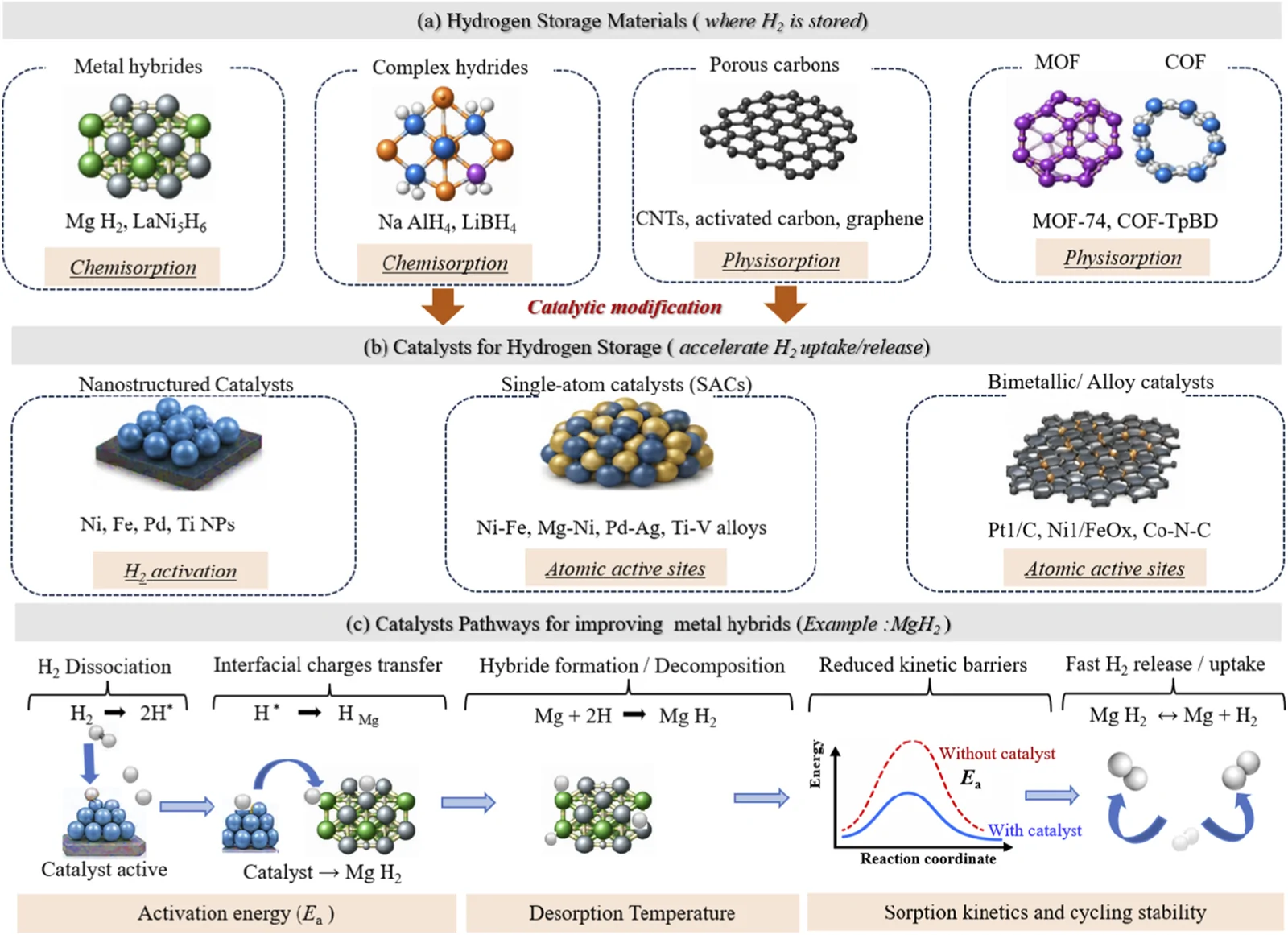
Schematic of the distinct roles in catalytic hydrogen storage: **(a)** storage materials that adsorb or chemically store H_2_; **(b)** catalysts that accelerate H_2_ activation and transport without storing it; **(c)** catalytic pathway for Mg H_2_ and the resulting performance gains.

A comparative overview of representation catalytic storage materials, including their hydrogen storage performance, catalytic mechanisms, key advances, and limitations, is presented in Table 1.

### 3.1 Classical materials (hydrides, porous carbons, MOFs)

Materials such as metal hydrides, porous carbon-based adsorbents, and MOFs are examples of conventional solid-state hydrogen storage options. In contrast to porous carbons and the majority of MOFs, metal hydrides mainly use chemisorption to store hydrogen. Several hydride systems, including magnesium hydride (MgH_2_), sodium aluminum hydride (NaAlH_4_), and complex hydrides doped with titanium, have been extensively studied for their favorable gravimetric capacities and thermodynamic characteristics (Boretti, 2025; Geb et al., 2024; Kalibek et al., 2024). For example, MgH_2_ has a theoretical hydrogen capacity of approximately 7.6 wt%. However, high desorption temperatures and slow sorption/desorption kinetics limit the practical use of many hydrides, as shown in Table 1. Substantial research has been undertaken to reduce activation barriers and improve hydrogen diffusion through catalytic doping with transition metals such as Ti, Ni, and Fe (Zhang X. et al., 2024; Wu J. et al., 2024). On the other hand, significant challenges related to cyclic stability and thermal control continue to hinder commercialization (Liu et al., 2024).

Activated carbon, carbon nanotubes, COFs, and graphene derivatives are examples of porous carbon materials that predominantly store hydrogen through physisorption (Thomas et al., 2024). Because of their structural tunability and large surface area, they show promise as reversible hydrogen adsorbents. However, their storage capacity at ambient temperature is generally limited, and substantial uptake commonly requires low temperatures or high pressures (Rampai et al., 2024). In these materials, molecular H_2_ is retained weak van der Waals interactions. Heteroatom doping can polarize H_2_ and strengthen adsorption, whereas supported metals amy promote dissociation and subsequent hydrogen spillover; however, excessively strong binding can impair reversibility.

Due to their structural tunability, variable pore sizes, and extraordinarily large surface areas, MOFs have been the subject of numerous studies as a potential material class for HS (Qiao et al., 2024; Letwaba et al., 2024). Composite formation can also improve the stability and gas-adsorption properties of MOFs. For example, incorporating MgMOF-74 into mesocellular foam enhanced its hydrothermal stability and preserved accessible adsorption sites, demonstrating the value of support assisted MOF design for gas-storage applications (Xin et al., 2021). MOFs such as MOF-5 and HKUST-1 can achieve substantial hydrogen uptake under cryogenic conditions (Qiao et al., 2024). Nevertheless, their limited host-guest interactions frequently lead to poor HS performance under variable temperature conditions. To that end, methods to improve hydrogen binding and spillover have been devised, including metal node engineering, linker functionalization, and the incorporation catalytic metals (Peng et al., 2024). Catalytic metals can promote H_2_ dissociation, while efficient spillover depends on catalyst-framework interaction, available receptor sites, and low interfacial migration barriers.

Collectively, classical materials provide the mechanistic and structural foundations for understanding HS… In light of these challenges, catalysis is crucial in bridging the gap between the theoretical and practical requirements of HS, enabling its practical consideration in the real world.

### 3.2 Advanced catalysts materials for HS

The development of advanced catalytic materials has emerged as an important strategy for overcoming the limitations of classical HSS, particularly regarding slow kinetic rates and the demanding operational conditions (Zhang X. et al., 2024; He et al., 2026). To improve overall storage performance, materials can be engineered at the atomic and nanoscale to optimize active-site density, electronic structure, and strength of hydrogen interactions. At the mechanistic level, these modifications regulate electron transfer and hydrogen-binding strength, thereby lowering the barriers for H_2_ dissociation, atomic hydrogen migration, and H_2_ recombination.

The use of metallic nanoparticles and nanocomposites as catalysts has also shown promising results for enhancing the processes related to hydrogen absorption and desorption (Zhang X. et al., 2024; Sun et al., 2024). Nanoscale engineering can increase surface area and active-site density while facilitating hydrogen dissociation and diffusion. Transition metals such as Ni, Fe, Pd, and Ti are commonly used because of their ability to activate molecular hydrogen. However, particle aggregation, oxidation, and structural degradation can reduce catalytic activity during repeated cycling. Nanoparticles provide abundant surface sites for H_2_ dissociation, while catalyst-host interfaces and nanoscale defects shorten hydrogen diffusion pathways. However, particle agglomeration reduces the accessible active site area and weakens these effects during cycling.

SACs represent recent achievements in catalytic design for HS. These systems consist of isolated metal atoms dispersed on supports such as carbon matrix, metal oxides, or porous structure. This configuration maximizes metal atom utilization and creates uniform, well-defined active sites (Chen et al., 2025; Liu et al., 2024). The kinetics for the storage reaction can be greatly enhanced, particularly in combination with hydride or porous host materials, by the thigh catalytic activities of SACs such as Pt_1_/C, Ni_1_/FeOx and Co-based systems, which are active for hydrogen dissociation (Liang et al., 2025; Zhang W. et al., 2024). The coordination environment of an isolated metal atom controls its local electronic structure and hydrogen-binding energy. Interaction among single atoms, support vacancies, and adjacent host species can facilitate H_2_ dissociation, interfacial hydrogen transfer and hydride decomposition. For example, Zhang et al. (Zhang J. et al., 2024) about reported a Ni_0.034_@TiO_2_ (SAC) for MgH_2_, whose addition at 15 wt% reduced the onset dehydrogenation temperature to 200 °C. At 300 °C, the resulting composite released 4.6 wt% H_2_ within 5 min and absorbed 6.53 wt% H_2_ within 10 s, while the apparent activation energies for dehydrogenation and hydrogenation decreased to 64.35 and 35.17 KJ mol^‒1^ H_2_, respectively. Furthermore, the composite retained 97.26% of its hydrogen storage capacity after 100 cycles. These improvements were attributed to the synergistic effects of isolated Ni atoms, oxygen vacancies, and multivalent Ti species, which promoted electron transfer and weakened Mg-H bonds (Zhang J. et al., 2024). In a related study, Lu et al. (Lu et al., 2025) investigated Mg H_2_ containing 5 wt% Cu SA/Ti_3_C_2_Tx, which exhibited an initial dehydrogenation temperature of 241.73 °C and a dehydrogenation activation energy of 76.38 KJ mol ^−‒1^ H_2_. After 20 cycles, the composite released 3.3 wt% H_2_ within 240 min at 180 °C. Despite these catalytic improvements, the relatively high catalyst loading can reduce the gravimetric hydrogen capacity of the composite. Moreover, maintaining the dispersion of isolated metal atoms and preventing their migration or aggregation during prolonged high-temperature cycling remain challenging. To achieve a balance between catalytic activity, structural stability, and overall hydrogen storage, more work is needed to create scalable and repeatable synthesis methods.

Bimetallic and alloy catalysts provide another strategy to enhance hydrogen interactions through synergistic electronic effects and lattice strain engineering. Systems such as Ni-Fe, Mg-Ni, Pd-Ag, and Ti-V exhibited tunable hydrogen binding energies compared with their corresponding monometallic materials (Yadav et al., 2025; Wu J. et al., 2024; Verma et al., 2023). These effects can reduce activation temperatures and enhance cycling behavior. For example, adding 5 wt% Al-Cu-Fe-Ni-Cr high entropy alloy to MgH_2_ decreased its initial desorption temperature from 425 °C to 180 °C. The composite absorbed 7.3 wt% H_2_ within 3 min and desorbed approximately 5.0 wt% within 6 min at 320 °C, while showing a capacity fluctuation of only approximately 0.05 wt% over 25 cycles (Yadav et al., 2025). Similarly, Mg H_2_ modified with Al_20_Cr_16_Mn_16_Fe_16_Ni_16_ absorbed approximately 6.1 wt% H_2_ within 2 min at 300 °C and 10 atm and released approximately 5.4 wt% within 40 min (Verma et al., 2023). On the other hand, problems such as phase segregation and compositional instability during repeated hydrogen cycling may impair long-term performance. Combining nano-structuring with alloy or composite formation can further improve catalytic activity (Zhang X. et al., 2024; Sun et al., 2024). A MgH_2_-5 wt% FeCoNiCrMn composite released 5.8 wt% H_2_ within 4 min at 300 °C and absorbed 5.5 wt% H_2_ within 0.5 min at 150 °C (Wan et al., 2023). Moreover, another CrMnFeCoNi-modified MgH_2_ composite released 6.5 wt% H_2_ with 10 min at 300 °C and began absorbing hydrogen at 40 °C (Wang et al., 2023).

Nanoscale alloy particles can promote hydrogen activation and diffusion kinetics through their synergistic electronic interactions and large surface area. Atomic reconstruction of Mg_2_Ni (Cu) also produced a reversible capacity of 6.1 wt% H_2_ with 95% capacity retention, demonstrating that suitable alloy reconstruction can improve catalytic activity and cycling stability (Zhang X. et al., 2024). Such integrated strategies may help balance catalytic activity, structural stability, and material cost. Modern catalysts allow HSS to work at lower temperatures, speed up reactions, and make them more reversible. On the other hand, improving the management of critical-material consumption, interactions between catalysts and storage materials, scalability in production, and durability is necessary for their practical application.

### 3.3 Emerging frameworks (COFs and hybrid composites)

Research into porous and hybrid frameworks for catalytic hydrogen storage is rapidly expanding. These frameworks combine complementary features such as large surface area, tunable surface chemistry, structural support, and catalytic activity. COFs and hybrid composite systems are particularly attractive because their compositions and architectures can be rationally tailored (Table 1).

Within COF crystals, light elements such as C, H, O, N, and B, are connected through strong covalent bonds. Their low density, ordered structures, and well-defined pore topologies can provide high thermal stability and structural tunability (Jiang et al., 2026; Côté et al., 2005). These characteristics make COFs promising hydrogen adsorbents, particularly under cryogenic conditions where physisorption dominates. Like other porous materials, COFs have low hydrogen binding energies at ambient temperature, which limits their effectiveness under practical operating conditions. Strategies such as metalation, catalytic-site incorporation, and polar-group functionalization have therefore been developed to enhance hydrogen interactions and promote spillover (Zhang W. et al., 2024). Mechanistically, metalation can polarize or dissociate H_2_, while polar groups strengthen host-guest interactions. Effective spillover additionally requires close catalyst framework interaction and low-energy pathways for atomic hydrogen migration. Using quantum-mechanical force fields and grand canonical Monte Carlo simulations, Djokic and Mendoza-Cortes showed that Co-, Ni-, Mn-, Fe-, Pd-, and Pt-containing multi-binding-site COFs could meet the target gravimetric and volumetric capacities at 298 K. The MSUCOF-3 family illustrated predicted total uptakes comparable to COF-301-PdCl_2_, which reached 60 gH_2_ L^‒1^ and 4.2wt% at 100 bar and 298 k (Djokic and Mendoza-Cortes, 2024). Also, new experimental evidence on carbon-based materials lends credence to this strategy. For example, at 298 K and 40 bar, respectively, a Pd Ni/microporous-carbon composite stored 0.95 and 1.22 wt% H_2_, while Ni Pd-modified reduced graphene oxide stored 2.65 wt% H_2_ (Chen et al., 2023). Graphene and microporous carbon, not COFs, were used to obtain these findings, and Pd makes materials more expensive. Although these modifications can increase the hydrogen uptake potential, experimentally measured room-temperature capacities remain below practical storage targets, and their long-term reversibility has not yet been sufficiently demonstrated. Beyond COFs, studies of carbazole-containing hydrogen-bonded organic frameworks have demonstrated how molecular building blocks and directional noncovalent interactions govern framework organization and electronic properties (Xia et al., 2024). Similarly, ion-framed porous organic polymers have shown that ionic functionality and tailored binding sites can regulate selective adsorbate-framework interactions. These findings offer more general principles for designing new porous frameworks to be functionalized, even though they were proved for non-hydrogen adsorbates (Ding et al., 2026). Hybrid composites combine two or more material classes to exploit their complementary properties (Wu J. et al., 2024; Sun et al., 2024). Composites of hydrides and carbon, carbons generated from MOFs, and nanoparticles embedded in porous frameworks are common architectures. One part of these systems may facilitate the activation of hydrogen, while another part supplies a host with a large surface area, structural support, or a network for transport (Wu J. et al., 2024; Jiang et al., 2026). For example, Ren et al. prepared MOF-derived nitrogen-doped porous nanofibers to support MgH_2_/Ni nanoparticles. The specific surface area of the carbon scaffold was 1,564.43 m^2^/g. In comparison to commercially available MgH_2_, the composite reduced the starting point for hydrogen release from 350 °C to 200 °C and maintained over 95.4% of its initial capacity following 10 cycles at 300 °C (Ren et al., 2022). The results of this work show that a carbon-supported hydride produced from MOFs has improved hydrogen-release performance and cycling stability.

Hybridization has the ability to deal with multiple constraints at once. A quantified example of hybridization was reported by Sun et al. Their MgH_2_-Ti_3_C_2_ MXene/graphene composite began releasing hydrogen at 169 °C and release 6.8wt% H_2_ within 6 min at 300 °C (Sun et al., 2024). Heat management and hydrogen transport can be improved by adding hydrides to thermally conductive frameworks. Enhanced cycling stability, reduced operating temperatures, and more usable capacity might result from these combined effects.

Despite these advantages, synthesis, consistency, scaling, and achieving a uniform distribution of active sites can be challenging with hybrid materials due to their structural complexity. Additional assessment under conditions relevant to their applications is necessary to understand how they behave during long-term absorption-desorption cycling (Jiang et al., 2026). COFs and hybrid frameworks offer diverse platforms. More accurate mechanistic understanding, scalable synthesis, repeatable structural control, and thorough evaluation of long-term durability are all necessary for their practical promise to be realized.

## 4 Recent breakthroughs in catalytic

Advances in nano-structuring, atomic-level catalyst design, and hybrid material engineering have led to important advances in catalytic HS in recent years. Improved mechanistic insight into hydrogen spillover, multifunctional hybrid systems, controlled defect engineering, nanoscale hydrides, and catalysts containing only one atom are some of the major advances. These developments have enabled more efficient hydrogen activation, improved thermodynamic properties, and faster reaction kinetics under milder operating conditions. These improvements reflect the transition from conventional materials discovery toward rational system design (Ma et al., 2025; Xu et al., 2024; Zhu et al., 2025).

One of the most promising developments is the emergence of SACs for HS SACs provide highly uniform active sites for hydrogen dissociation because isolated metal atoms are distributed on appropriate supports, allowing for efficient utilization of the metal atoms (Liang et al., 2025; Zhang W. et al., 2024). As a result, hydrogen uptake and release can be substantially improved, especially when SACs are utilized in conjunction with hydrides and/or porous host materials (Hussain et al., 2024). An ongoing problem in the design of storage materials is achieving a balance between efficient hydrogen activation and reversible desorption; the electronic control of discrete SAC active sites provides a promising strategy for addressing this trade-off (Ma et al., 2025).

Another important area of development involves defect engineering and nanoscale control. Carbon nanotubes and metal-organic frameworks are examples of porous materials that can have their number of active sites increased and hydrogen spillover facilitated through the presence of vacancies, heteroatom sites, and coordinatively unsaturated sites. Nanoparticles and artificially generated defects in metal hydrides can decrease desorption temperatures by shortening the hydrogen-diffusion pathways. Excessive structural disorder, however, can reduce cycling stability; hence, defect concentration needs to be carefully regulated.

A growing number of researchers are focusing on hybrid catalytic systems as a means to of enhancing HS performance by integrating multiple functional components. To, achieve synergistic effects, these systems may incorporate carbon matrices, metal nanoparticles, hydrides, or metal-organic frameworks. MOF-derived carbon materials incorporating transition-metal nanoparticles (e.g., CoNi or FeNi) can exhibit important electrical conductivity, structural stability, and catalytic activity. These multifunctional designs provide a promising strategy for accelerating hydrogen sorption and improving cycling performance (Hu et al., 2025). In these hybrid systems, metal sites dissolve molecular hydrogen, interfaces facilitate atomic hydrogen transfer, and porous hosts offer accessible storage sites. These cooperative effects can improve hydrogen capacity, accelerate adsorption and desorption, and enhance cycling stability.

Significant progress has also been achieved in clarifying the mechanism of hydrogen spillover. Computational studies and advanced characterization techniques have improved our understanding of dissociated hydrogen atom transport from catalytic sites to storage hosts. This knowledge enables the rational design of catalyst–storage interfaces to enhance hydrogen transfer efficiency. Recent research on Pt@MOF-801 demonstrated that MOF-based composites can benefit from water molecules enabling hydrogen migration (Gu et al., 2023). In addition, studies incorporating metal nanoparticles into HKUST-1-type MOFs demonstrated that the enhancement of HS capabilities can be achieved by the utilization of electron transport effects caused by spillover (Qiao et al., 2024; Peng et al., 2024; Letwaba et al., 2024; Gu et al., 2023).

These findings identify interfacial water, charge transfer, and catalyst-host interactions as important variables in catalytic hydrogen storage. Underground hydrogen storage has also made use of catalytic and interfacial concepts. Hydrogen mobility and retention in porous geological formations can be impacted by mineral surfaces via adsorption, surface complexation, and geochemical processes. Mineral surface charge, pore structure, and geochemical circumstances can impact hydrogen behavior, according to recent investigations of sandstone reservoirs (AlDhuhoori et al., 2025). As stated in another experiment, the circumstances in the reservoir under investigation may limit the abiotic interactions that can occur between hydrogen and sandstone (Hassanpouryouzband et al., 2022). However, evidence for beneficial mineral-surface catalysis remains preliminary, and further investigation is required to determine whether these reactions improve hydrogen retention or cause hydrogen loss and contamination.

New research has shown that several approaches have the potential to improve HS performance. These include controlled defects, multifunctional surfaces, nanoscale hydrides, atomically dispersed catalysts, and spillover-pathway engineering. Storage capacity, sorption rate, operating temperature and pressure, catalyst loading, reversibility, and capacity retention are some of the characteristics that should be reported in standardized methods in future investigations. Additional issues that need to be resolved before commercial application include scalable synthesis, degradation of catalysts, stability of interfaces, and long-term safety.

## 5 Mechanistic insights and theoretical advances

A more in-depth understanding of HS processes at the molecular and atomic levels is crucial for guiding the rational design of next-generation catalytic materials. Mechanistic insights are essential for establishing relationships among material structure, surface chemistry, and macroscopic storage performance. Theoretical and computational approaches are employed to predict and optimize catalytic activity, particularly when experimental data are limited (Zhang J. et al., 2024; Chen et al., 2025; Zhou et al., 2024).

Catalytic hydrogen sorption generally begins with the adsorption and activation of molecular hydrogen (H_2_), typically followed by its dissociation on catalyst surfaces. At this stage, the electronic structure of catalyst plays a significant role, especially for transition metals, because of the availability of d-orbitals for (H-H bond) cleavage (Zeng et al., 2024). The dissociated hydrogen atoms migrate across the surface of the catalyst and into the storage medium (Fareed et al., 2025). Within metal hydrides, hydrogen subsequently diffuses through the lattice and participates in hydride nucleation and growth. In porous adsorbents, hydrogen remains primarily associated with surfaces and pores through weak interactions, although catalytic sites may strengthen adsorption locally. The effectiveness of these processes greatly influences the overall reversibility and kinetics of HSSs (Chen et al., 2025). The rate-limiting step may involve H_2_ dissociation, interfacial transfer, bulk diffusion, hydride formation, or H_2_ recombination, depending on the material and operating conditions. Importantly, catalysts primarily lower kinetic barriers and accelerate the approach to equilibrium; they do not necessarily alter the equilibrium thermodynamics or theoretical hydrogen capacity of the storage material.

Spillover of hydrogen from metal catalysts to porous supports, such as carbon materials, MOFs, or COFs, is one of the most extensively studied phenomena in composite systems (Shen et al., 2022; Shun et al., 2022). Hydrogen atoms are released onto the surrounding support surface after hydrogen molecules dissociate at metallic catalytic sites, a process known as hydrogen spillover. The overall process comprises three principal steps: H_2_ dissociation at the catalyst, transfer of atomic hydrogen across the catalyst-support interfaces, and migration among receptor sites on the support. During desorption, reverse spillover may occur as hydrogen atoms return to catalytic sites and recombine to form H_2_. In addition to the HS sites offered by the metal catalyst, this process can increase the number of available adsorption sites. Despite experimental evidence of spillover in several material systems, the exact extent of its contribution remains under debate since it is difficult to isolate and quantify independently of other hydrogen transport and adsorption mechanisms. Measured uptake may also be influenced by defects, residual solvents, moisture, pore filling, and experimental conditions, which complicates the assignment of capacity enhancements solely to spillover. However, careful design of the catalyst-support interface is considered crucial for promoting hydrogen migration and improving spillover efficiency. Short diffusion distances, strong catalytic-support interaction, suitable receptor sites, and more moderate hydrogen-binding energies can facilitate interfacial transfer while maintaining reversible desorption.

Electronic interactions between catalysts and supports, activation barriers, reaction pathways, hydrogen adsorption energies, and charge transfer are effectively investigated using density functional theory (DFT) (Wu et al., 2019). Parameters that are typically generated from DFT include activation-energy barriers, density of states, charge-density differences, Bader charge transfer, and hydrogen adsorption and binding energies. Preferred adsorption sites, catalyst-hydrogen interactions, and electronic changes related to H_2_ activation can be better understood through these parameters. DFT calculations can also construct potential-energy profiles for adsorption, dissociation, surface migration, interfacial transfer, bulk diffusion, and recombination. These profiles help identify the rate-limiting elementary step and determine how alloying, coordination, strain, vacancies, and support interactions modify catalytic activity. An optimal catalyst should bind hydrogen strongly enough to promote H_2_ dissociation but weakly enough to permit hydrogen migration and reversible release.

To assess possible materials, DFT simulations can predict hydrogen binding strength and identify the optimal electronic configurations for catalytic activity. The structural stability, adsorption energy, electronic structure, and desorption conditions were examined in a DFT study of Mg-modified C_7_N_3_ material, which demonstrated reversible HS behavior (Duan et al., 2025). Additionally, spin-polarized DFT simulations were used to study defect production and transition metal doping in MoSe_2_/graphene. The results showed that Ni and Cu doping altered the electronic structure and adsorption properties are changed, leading to an enhanced H_2_ absorption (Alfalasi et al., 2024). This research demonstrated that DFT can support the methodical creation of materials with favorable hydrogen-binding properties. Theoretical research is also useful for liquid organic hydrogen carrier systems. A recent DFT analysis of the hydrogenation of N-ethyl-carbazole over supported Ni_4_ clusters clarified how the metal structure and electron-rich sties influence hydrogen adsorption and reaction pathways (Zhou et al., 2024).

To complement static DFT investigations, molecular dynamics (MD) simulations are employed to evaluate structural stability, temperature-dependent behavior, and hydrogen diffusion (Yang et al., 2024). Dynamic characteristics such the mean square displacement, hydrogen diffusion coefficient, radial distribution function, coordination number, interaction energy, residence duration, and temperature-dependent molecular mobility are provided by MD simulations. Under operational conditions, these characteristics characterize structural evolution, local interactions, and hydrogen transport.

Hydrogen migration in porous framework materials and elemental phase transformations in hydride materials during cycling are two examples of dynamic processes that can be studied more effectively using MD simulations (Oliver et al., 2024). Reactive MD can additionally capture bond-breaking and bond-forming events, whereas conventional MD is more suitable for diffusion, structural evolution, and thermal stability. However, the reliability of MD predictions depends strongly on the selected force field and its ability to represent catalytic-hydrogen and host-hydrogen interactions. DFT and MD are complementary components (Chen et al., 2026). DFT provides electronic and elementary-reaction information, while MD extends the analysis to larger spatial and temporal scales. Kinetic Monte Carlo and continuum models can further connect atomistic events with particle-level sorption rates and system-scale transport.

Recent efforts to expedite catalyst discovery have also employed data-driven modeling and machine learning (ML). Using the current experimental and computational data, ML models can be constructed to rapidly predict potentially synthesizable material compositions (Osman et al., 2024c; Ahmed and Siegel, 2021). Consequently, large chemical spaces can be investigated more effectively, and catalyst synthesis requires less reliance on trial and error (Khatamirad et al., 2023; Goldsmith et al., 2018). Parameters such as adsorption energy, activation barrier, metal coordination, defect formation energy, pore size, surface area, and hydride formation enthalpy can be used to relate material structure to storage performance. However, inconsistent experimental conditions and limited high-quality datasets can reduce model transferability and predictive reliability. Despite these advances, major gaps remain in existing mechanistic knowledge. The effects of defects at catalyst support interfaces on hydrogen migration and charge transfer remain insufficiently understood (Zhu et al., 2025; Shen et al., 2022). More accurate modeling conditions that account for structural disorder, temperature, and pressure are necessary to address discrepancies between experimental results and theoretical expectations (Khatamirad et al., 2023). For example, solvent molecules, surface oxidation, catalyst restructuring, particle agglomeration, and phase evolution during cycling should also be considered because idealized static models may not represent working storage systems. Operando spectroscopic and microscopic techniques are therefore needed to identify active sites, monitor intermediate species, and validate predicted reaction pathways under realistic conditions. Research in HS is moving away from material-driven screening and toward rational design and prediction as a result of advances in modeling and mechanisms understanding. To create effective and practical catalytic HSSs, it is essential to combine computational modeling with experimental validation (Osman et al., 2024c; Nørskov et al., 2009).

## 6 Challenges and future perspectives

Catalytic HS materials have advanced significantly; however, fundamental and practical challenges remain. The central challenge differs by material class. For Mg-based hydrides, catalysts can accelerate hydrogen dissociation and diffusion, but the heat required for hydrogen release remains a major constraint. For porous adsorbents, weak hydrogen binding limits uptake near ambient temperature, even when adsorption and desorption are readily reversible. Moreover, MOFs, COFs, and porous carbons should therefore be evaluated by their usable capacity across to defined pressure range and the cooling energy required to achieve that capacity.

Repeated hydrogen absorption/desorption poses a significant obstacle to the durability of catalytic systems. Structural of deterioration, particle agglomeration, alloy phase separation, and pore collapse of porous can have a significant impact on performance during cycling. Furthermore, catalyst poisoning and surface oxidation can eliminate active sites. The effects of realistic gas impurities remain insufficiently studied; cycling tests should compare high-purity H_2_ with gas containing measured levels of relevant impurities, such as water, oxygen, and carbon monoxide. These mechanisms should be investigated separately for each other material. For hydride catalyst composites, researchers should determine whether capacity loss arises from particles coarsening, changes in the hydride phase, or reduced contact between the catalyst and hydride. For porous frameworks and supported single-atom catalysts, studies should track pore integrity, metal coordination, and active-site retention before and after cycling.

Catalyst deactivation can occur even when the storage material retains its original crystal structure. Particle sintering or migration, reduces the surface available for hydrogen dissociation. Oxides layers and impurities block active sites, while reactions between the catalyst and hydrogen may form fewer active phases. In SACs, changes in metal coordination or aggregation into clusters can eliminate the intended isolated sites. Comparing fresh and cycled materials through microscopy, phases and surface analysis, and H_2_ uptake and release tests can identify these changes. So, studies should report both capacity retention and reaction rates, since kinetic activity may decline before storage capacity does.

Scalability and cost further limit commercialization. Precisely engineered nanomaterials developed can be costly to produce, and while noble metal catalysts perform well in the laboratory, their high cost and limited availability restrict their use at the scale required for industrial production. In addition, the reproducibility of synthesis procedures for sophisticated materials including MOFs, COFs, and single-atom catalysts remain challenging, which makes their commercial deployment more difficult.

A promising result obtained with a small powder sample should be tested in a larger, packed storage bed, where heat transfer, gas flow, packing density, and the mass of the supporting framework affect usable capacity and release rate. Further studies should include a techno-economic analysis that accounts for catalyst loading, precursor and synthesis costs, material yield, storage-bed components, heating or cooling energy, and replacement costs over the system’s service life. Sensitivity analysis should identify which factors most strongly affect the cost per kilogram of usable H_2_ delivered.

Materials selection and reactor design also determine safety. For example, hydrides that release substantial heat during charging require effective heat removal, while materials reactive toward air or moisture require appropriate containment. Reactor designs should account for the material’s operating pressure and temperature, provide pressure relief and leak detection, and maintain safe conditions during charging, discharge, and shutdown.

An inadequate understanding of the interaction pathways at complex surfaces hinders the rational design of new HS materials from a mechanistic standpoint. Diverse difference between experimental results and theoretical predictions remain particularly regarding hydrogen spillover, interfacial charge transfer, and defect-mediated diffusion. Operando X-ray diffraction can track hydride phase changes, while X-ray absorption spectroscopy can reveal changes in catalyst oxidation state and coordination. Neutron diffraction can locate hydrogen in a hydride, and neutron imaging can identify uneven uptake across a storage bed. Catalyst-free and support-only controls can help distinguish material changes from catalyst-interface effects. Models should use experimentally relevant temperatures, pressures, and material structures, then predict observable quantities such as reaction rates or adsorption behavior. Furthermore, the discovery of inexpensive catalytic materials composed of earth-abundant elements, is essential for the economic viability of industrial scale operations. To accelerate the discovery of improved catalytic systems, data-driven technologies such as machine learning and high-throughput computational screening could be utilized. The primary objectives of these approaches are to discover promising material combinations more rapidly, reduce reliance on trial-and-error experimentation, and minimize the effort required at each stage of material development.

## 7 Conclusion

The slow sorption kinetics and many HS materials can be improved through catalytic approaches, although low volumetric energy density remains a challenge at the system level. Advancements in metal hydrides, porous materials, covalent organic frameworks, nanostructured catalysts, single-atom catalysts, bimetallic alloys, and hybrid composites are highlighted in this mini-review. Enhanced reversibility, reduced activation barriers, and accelerated hydrogen uptake and release are potential benefits of catalytic engineering applied to these materials.

The simultaneous optimization of kinetics, cycling stability, reversibility, and storage capacity has become a central priority in materials design. Atomic-scale catalyst design at the atomic scale, nanoscale engineering, and hybrid architectures have all contributed to improved interfacial transport and hydrogen activation. Nevertheless, achieving high H_2_ capacity, fast kinetics, long-term stability, and near-ambient operation within a single system remains difficult. Bridging the gap between laboratory scale performance and practical deployment therefore remains critical objective.

Machine learning-assisted materials discovery, improved interface optimization, realistic multiscale modeling, enhanced *in situ* characterization, and defect engineering are all necessary for future advancements. Future work should assess performance under realistic operating and gas-composition conditions, alongside reactor safety, scalable synthesis, and cost. For the development of practical next-generation HS technologies, it is vital that computational modeling, materials chemistry, and catalysis continue to be integrated. Such integration will support the rational development of efficient, durable, scalable, and economically viable catalytic HSS.
